# RAGE controls leukocyte adhesion in preterm and term infants

**DOI:** 10.1186/s12865-014-0053-0

**Published:** 2014-11-27

**Authors:** Kirsten Buschmann, Raphaela Tschada, Marie-Sophie Metzger, Natascha Braach, Navina Kuss, Hannes Hudalla, Johannes Poeschl, David Frommhold

**Affiliations:** Department of Neonatology, University Hospital, 69120 Heidelberg, Germany

**Keywords:** Fetal, Preterm, Neonate, Neutrophils, Leukocyte adhesion, RAGE, Inflammation

## Abstract

**Background:**

Insufficient leukocyte recruitment may be one reason for the high incidence of life-threatening infections in preterm infants. Since the receptor of advanced glycation end products (RAGE) is a known leukocyte adhesion molecule and highly expressed during early development, we asked whether RAGE plays a role for leukocyte recruitment in preterm and term infants.

**Methods:**

Leukocyte adhesion was analyzed in dynamic flow chamber experiments using isolated leukocytes of cord blood from extremely premature (<30 weeks of gestation), moderately premature (30–35 weeks of gestation) and mature neonates (>35 weeks of gestation) and compared to the results of adults. For fluorescent microscopy leukocytes were labeled with rhodamine 6G. In the respective age groups we also measured the plasma concentration of soluble RAGE (sRAGE) by ELISA and Mac-1 and LFA-1 expression on neutrophils by flow cytometry.

**Results:**

The adhesive functions of fetal leukocytes significantly increase with gestational age. In all age groups, leukocyte adhesion was crucially dependent on RAGE. In particular, RAGE was equally effective to mediate leukocyte adhesion when compared to ICAM-1. The plasma levels of sRAGE were high in extremely premature infants and decreased with increasing gestational age. In contrast, expression of β_2_-Integrins Mac-1 and LFA-1 which are known ligands for RAGE and ICAM-1 did not change during fetal development.

**Conclusion:**

We conclude that RAGE is a crucial leukocyte adhesion molecule in both preterm and term infants.

**Electronic supplementary material:**

The online version of this article (doi:10.1186/s12865-014-0053-0) contains supplementary material, which is available to authorized users.

## Background

During the past decades advances in neonatal medicine led to strongly improved survival of extremely premature infants [1]. Nonetheless, especially among very immature infants, infection and sepsis are still the leading causes for mortality and morbidity [2-4]. As immaturity of the innate immune system seems to be one of the reasons for this observation, the role of leukocytes has been addressed by numerous studies [2-5]. In particular, fetal leukocyte recruitment has been increasingly examined both in vitro and in vivo [6-8].

The cascade of leukocyte recruitment plays a crucial role in the immune defense during inflammation [9]. Capture of free flowing leukocytes is followed by leukocyte rolling along the endothelial layer, triggering the activation of β_2_-integrins, i.e. LFA-1 (CD 11a/CD18) and Mac-1 (CD 11b/CD18), which interact with different endothelial ligands such as ICAM-1 [10,11]. This leads to firm adhesion to the inflamed endothelium and finally to leukocyte transmigration [9,12].

Recently, Nussbaum et al. and Sperandio et al. investigated leukocyte recruitment during fetal development using flow chamber experiments in humans and a new fetal mouse model [6,8]. They showed that fetal leukocyte recruitment matures during pregnancy, which may account for the high susceptibility of preterm infants to invasive infections. Consistent with other studies, they also observed that the expression of leukocyte rolling molecules, L- and P-selectin and P-selectin-glycoprotein-ligand 1 and adhesion molecules ICAM-1, Mac-1 and IL-8-receptor increase with gestational age [6,8,13-18].

The receptor of advanced glycation end products (RAGE), a strong activator of nuclear factor kB (Nf-kB) [19], is highly expressed during fetal development [20,21]. RAGE plays a crucial role in a variety of inflammatory diseases [22-25]. Beside its signaling function, RAGE serves as a multiligand receptor, binding to high-mobility group box 1 protein, protein S100, Mac-1 and others [10,11,26-29]. Interestingly, RAGE was also discovered to mediate leukocyte adhesion via direct binding to Mac-1 [10,11].

Soluble RAGE (sRAGE) is formed by shedding of the receptor’s extracellular domain and therefore lacks intracellular signaling. Thus, sRAGE may serve as a decoy receptor that may antagonize functions of full-length RAGE [22,23]. Only very few studies investigated sRAGE-expression in extremely premature infants. Similar to inflammatory conditions in adults they showed decreased fetal sRAGE concentrations in the presence of chorioamnionitis [30,31]. The high fetal RAGE expression indicates a pivotal role of RAGE during early development. However, its function for fetal leukocyte recruitment remains unclear.

Therefore, we investigated RAGE-dependent leukocyte adhesion in premature and term infants at different gestational ages.

## Methods

### Sample collection and study population

All included infants were delivered by primary cesarean section. 5-10 ml of umbilical cord blood was collected immediately after delivery. Children with severe fetal malformations, infectious maternal diseases (i.e. chorioamnionitis) and familial immune diseases were excluded. In addition, 10 ml of peripheral venous blood from healthy adult volunteers was drawn by venipuncture. Standard blood collection tubes (S-Monovette, Sarstedt, Nümbrecht, Germany) containing trisodiumcitrate were used for anticoagulation. Based on their gestational age, as estimated by the date of the last menstrual period and by ultrasound measurements, infants were grouped into extremely premature infants (<30 completed weeks of gestation), moderately premature infants (30–35 weeks of gestation), and mature neonates (>35 completed weeks of gestation). Infants older than 35 gestational weeks were considered to be immunologically mature. Informed, written consent was obtained from all adult volunteers and all mothers whose children were included in our study. The study was approved by the local Medical Ethical Committee of the Ruprecht-Karls-Universität (S-047/2008).

### Isolation of polymorph-nuclear leukocytes (PMNs)

As fetal leukocyte and differential white blood counts may vary largely depending on the gestational age, we isolated and quantified PMNs from the umbilical cord of neonates, premature infants or peripheral venous blood of healthy adults. Whole blood was layered onto a density gradient (LSM 1077; PAA Laboratories GmbH, Coelbe, Germany) and centrifuged (1200 × *g*, 20 min, 4°C). The resulting erythrocyte-granulocyte pellet was washed twice in Dulbecco’s PBS (1x, without Ca^++^ and Mg^++^; Invitrogen GmbH, Darmstadt, Germany) and erythrocytes were lysed by hypotonic buffer (0.15 M NH_4_Cl, 0.01 M NaHCO_3_^−^, 0.001 M EDTA, in Aqua ad injectabilia for 7 min in the dark at room temperature). The remaining cells were washed twice, resuspended in 1 ml PBS, and counted in a Neubauer chamber (Bright-Line®, Hausser Scientific, Horsham, PA, USA) using Turks solution (Merck, Darmstadt, Germany). The number of PMNs, lymphocytes, erythroid and myeloid precursors was then quantified by May Gruenwald staining (Merck, Darmstadt, Germany) using known cytomorphological parameters [7,32,33]. For additional cell differentiation we performed flow cytometry using standard leukocyte clusters defined by forward-side scatter analysis. To identify PMNs during subsequent flow chamber experiments leukocytes were stained with the fluorescent dye Rhodamine 6G (max 10 min) which does not stain erythroid cells [34,35].

### Preparation of murine sRAGE

Mouse sRAGE was kindly provided by Prof. Peter Nawroth (Dept of Medicine I & Clinical Chemistry, University Heidelberg). The preparation is summarized in brief. A plasmid with the coding sequence of the mouse extracellular domain of RAGE (1030 bp) was cloned into pET-DEST42 (Invitrogen, Darmstadt, Germany) and transformed into the *Escherichia coli* strain BL21. Next, isopropyl D-thiogalactopyranoside induced soluble RAGE (sRAGE) protein expression, which was purified using Protino Ni-TED 2000 columns (Macherey-Nagel, Dueren, Germany). Finally, potential endotoxin contamination was removed by affinity chromatography EndoTrap blue 5/1 (Profos AG, Regensburg, Germany).

### sRAGE ELISA

sRAGE concentrations in citrated plasma were measured with a commercially available sandwich enzyme-linked immunosorbent assay (Biovendor, Modrice, Czech Republik) according to the manufacturer’s instruction. This assay is known to specifically detect sRAGE in human plasma. Finally the extinction was determined with a Flashscan microplate reader (Analytik Jena AG, Jena, Germany) at 450 nm.

### Flow cytometry

The expression of Mac-1 and LFA-1 was assessed by flow cytometry as described previously [11]. After red blood cell lysis, 10^5^ cells were incubated in the dark with 2 μg FITC-conjugated anti-LFA-1 or anti-Mac-1 (eBioscience, San Diego, USA) or 2 μg FITC-conjugated isotype control antibodies (Mouse IgG1, eBioscience, San Diego, USA). Mac-1 and LFA-1 expression was assessed on 10.000 cells per mouse within the neutrophil cluster defined by forward-sideward scatter analysis using LSRII with DIVA software package (Becton Dickinson, San Jose, USA). Expression of Mac-1 and LFA-1 was compared to their respective isotype controls.

For differentiation of cells before and after isolation procedures flow cytometry was performed on 10^5^ unstained cells using standard neutrophil, monocyte and lymphocyte clusters defined by forward-sideward scatter analysis.

### Flow chamber experiments

Flow chamber experiments were conducted as described [36,37]. In brief, rectangular microglass capillaries (VitroCom, Mountain Lakes, USA) were coated with rh P-selectin (4 μg/ml, R&D Systems, Wiesbaden, Germany), rh CXCL8/IL-8 (10 μg/ml, Peprotech, London, United Kingdom), and rh ICAM1 (4 μg/ml, R&D Systems) or sRAGE (4 μg/ml or as indicated) and connected via PE tubing to a 2 ml syringe containing freshly isolated neutrophils. Due to the high number of erythroid progenitors in some groups, cell suspension was then incubated with the fluorescent dye Rhodamine 6G (75 μl/10^6^ cells/ml) for leukocyte staining. The number of neutrophils was now set at 2×10^6^ cells/ml by counting fluorescent cells in a Neubauer chamber by fluorescent microscopy using the FITC channel (BX51 WI , with a saline immersion objective × 20/0.95 NA, Olympus Hamburg). The cell suspension was perfused through the flow chamber and neutrophil adhesion was observed by fluorescent microscopy for 10 minutes under constant flow conditions using a high precision perfusion pump (Harvard Instruments, March-Hugstetten, Germany; wall shear stress 0.1 Pa). Images were recorded via a CCD camera system (CF8HS; Kappa) on a Panasonic S-VHS recorder. Permanent adherent fluorescent cells were counted as neutrophil adhesion per field of view (FOV) after 10 min.

### Statistics

Sigma Stat 3.5 (Systat Software, Erkrath, Germany) was used for statistical analysis. Clinical and laboratory parameter of patients, leukocyte adhesion, sRAGE concentration and LFA-1 and Mac-1 expression between groups were compared with one-way ANOVA followed by a multiple pairwise comparison test (Dunn’s test) or by Wilcoxon rank-sum test, as appropriate. Statistical significance was set at *p* < 0.05 or as indicated.

## Results and discussion

### Study population

76 infants were included in our study from 7/2008 to 5/2012. The participants consisted of mature infants (>35 gestational weeks, n = 50), moderately premature infants (30–35 gestational weeks, n = 14) and extremely premature infants (<30 gestational weeks, n = 12). In addition, we analyzed blood samples of 29 healthy adult volunteers (male 13: female 16; mean age = 27.6 ± 6.8 years). Reasons for prematurity were placental insufficiency, pre-eclampsia, HELLP syndrome, pathologic Doppler flow, and twin pregnancy. Children with underlying infectious diseases of the mother, e.g. suspected chorioamnionitis were excluded. The patient characteristics and laboratory data are shown in Table 1.

**Table 1:** **Patient characteristics and laboratory values of umbilical cord blood**

**Data**	**Extremely premature**	**Moderately premature**	**Mature neonates**	**p value**
	**(<30 weeks)**	**(30–35 weeks)**	**(>35 weeks)**	**(ANOVA)**
Number	n = 12	n = 14	n = 50	
***Clinical***				
GA (weeks)	28 4/7 ± 1 4/7	33 1/7 ± 1 1/7	37 3/7 ± 1 2/7	na
Birth weight (g)	1130 ± 260*^#^	1930 ± 570*	3040 ± 520	<0.001
Female/male	4/8	6/8	23/27	na
APGAR 5ʹ	8 ± 1	9 ± 1	9 ± 1	ns
10ʹ	8 ± 1	9 ± 1	9 ± 1	ns
***Laboratory***				
Arterial pH	7.31 ± 0.07	7.30 ± 0.04	7.28 ± 0.05	ns
CrP (mg/dl)	<0.05	<0.05	<0.05	na
WBC/nl	9.3 ± 3.6*	12.3 ± 3.9	14.5 ± 7.9	<0.05
PMN (%)	25.4 ± 12.8*	32.4 ± 9.5*	45.5 ± 14.2	<0.001
Hct (%)	0.45 ± 0.05	0.50 ± 0.04	0.48 ± 0.06	ns

Data are given as mean ± SEM if applicable. GA, gestational age; APGAR score; WBC, White blood cell count; Hct, haematocrit; CrP, C-reactive protein; na, not applicable; ns, not significant. The asterisk (*) indicates significant differences vs. mature neonates and the pound key (^#^) vs moderately premature infants as given by the P value (ANOVA).

Since children were included consecutively, the gender distribution among the experimental groups shows some variation. As expected, premature infants had a significantly lower birth weight than mature neonates. In addition, white blood-cell count and PMN count in whole blood were significantly lower in premature than in mature infants, an observation that has also been described earlier [6]. Notably, levels of C-reactive protein did not vary between investigated groups. Prenatal steroids (betamethasone) were administered to the majority of mothers of preterm infants. Although steroids are discussed to alter neutrophil function, recently Nussbaum et al. were able to exclude an impact of betamethasone on leukocyte adhesion using the same setting of flow chamber experiments as we did in our study [6]. Therefore, we argue that prenatal steroid administration should not influence leukocyte adhesion in our study.

### Age dependent characteristics of PMN isolates

**Figure 1:**
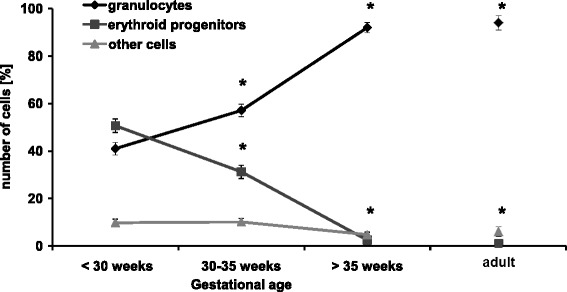
**Gestational age dependent distribution of blood cells after PMN isolation procedure.** Granulocytes, erythroid progenitors and other cells (lymphocytes and myeloid precursors) from extremely, moderately premature and mature infants as well as adults were counted using differential May Gruenwald staining (mean ± SEM). Significant differences to extremely premature infants (<30 weeks of gestation) are indicated by asterisks (p <0.05).

Since we aimed to specifically investigate adhesion behavior of neutrophils, we tested different PMN isolation procedures and evaluated the resulting purity of the cell suspension. As standard PMN isolation methods using dextrose density gradient centrifugation failed in newborns, we performed an inverse separation with LSM 1077, which primarily isolates lymphocytes. After May-Gruenwald staining of the resulting cell suspension we counted about 90% PMN in mature newborns compared to 92% PMN in adults (Figure 1, see also Additional file 1: Figure S1).

These results were confirmed by flow cytometric counts of cell populations using standard gates defined by forward-side scatter analysis: after isolation PMNs increased to 93% (<1% monocytes and 7% lymphocytes or others) in adults and to 88% (<1% monocytes and 12% lymphocytes or others) in term infants (Additional file 1: Figure S2 and Figure S3). However, after the described PMN isolation procedure we found only 60% PMNs in moderately premature infants and only 40% PMNs in extremely premature infants (Figure 1). According to differential staining, the remaining cells were mostly erythroid progenitor cells (30% and 50%, respectively) and, less prominent, lymphocytes and myeloid precursors, summed up as others (10%) at these gestational ages (Figure 1). In contrast, erythroid progenitor cells, lymphocytes and myeloid precursors were hardly found in term infants and adults after isolation (about 10% and less, Figure 1).

These results are in line with previous studies, demonstrating that the number of circulating erythroid progenitor cells is particularly high in premature infants and that they are difficult to separate from leukocytes due to physical similarities [38-40]. Although these cells have been shown to induce immunosuppression in neonates they should not alter leukocyte adhesion in our flow chamber experiments since they need the whole organism for interaction [38-40]. To answer the question whether erythroid progenitor cells might directly mimic neutrophil behavior during flow chamber experiments, it is important to mention that they only express PSGL-1, but not IL-8 receptor, Mac-1 nor LFA-1 which are crucial adhesion molecules in this setting [9,12,41,42]. Thus, it is unlikely that erythroid progenitor cells adhere to flow chambers coated with P-selectin, IL-8 and ICAM-1/RAGE.

However, to exclude varying neutrophil count in cell isolates of different age groups and misinterpretation of adherent cells, all cell isolates were further treated with the fluorescent dye Rhodamine 6G which predominantly stains leukocytes, while erythroid cells remain unstained [34,35]. Using fluorescent microscopy we were now able to set the number of neutrophils at 2×10^6^/2 ml suspension in all age groups and to specifically analyze neutrophil adhesion in flow chamber experiments.

### In-vitro neutrophil adhesion in preterm and term infants

**Figure 2:**
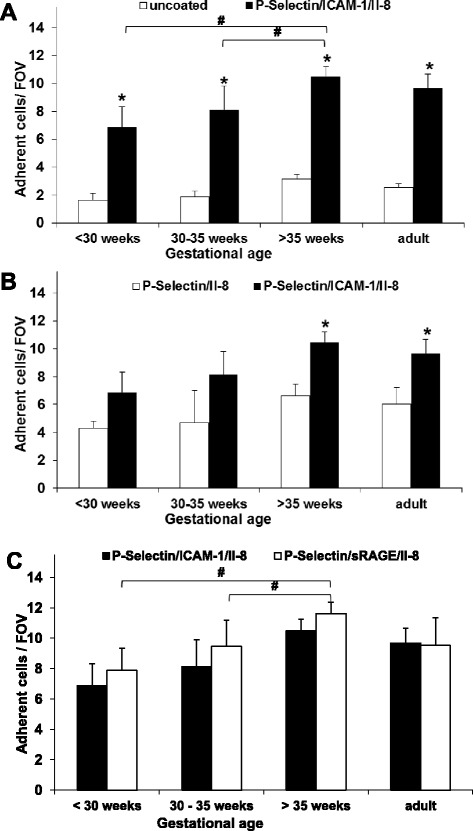
**RAGE- and ICAM-1-dependent fetal neutrophil adhesion.** Neutrophil adhesion of preterm and term infants and adults was analyzed in dynamic microflow chamber experiments **(A)** Neutrophil adhesion upon P-selectin, IL-8 and ICAM-1 was compared between the various age groups and to respective uncoated controls. **(B)** Neutrophil adhesion is shown for P-selectin and IL-8 coated flow chambers with and without ICAM-1. **(C)** ICAM-1-and sRAGE-dependent neutrophil adhesion in combination with P-selectin and IL-8-coating. Results are presented as mean + SEM from at least 5 separate individuals/experiments per group. Significant differences (p <0.05) to uncoated or P-selectin and IL-8 coated flow chambers are indicated by asterisks and by the pound key (as indicated).

In order to investigate the role of RAGE for fetal leukocyte recruitment, we measured adhesion of neutrophils of adults, term and premature infants in dynamic microflow chambers experiments (see also representative screenshot in Additional file 1: Figure S4). Different coating of flow chambers allowed a functional analysis of the used or omitted molecules. As reported previously [37,43], we first used a combination of P-selectin, IL-8 and ICAM-1, which triggers significant adhesion of adult neutrophils when compared to uncoated controls (Figure 2A). This molecular coating also led to a relevant adhesion of neutrophils of neonates or premature infants compared to their respective controls. However, the number of neutrophils that adhered on P-selectin, IL-8 and ICAM-1 gradually and significantly increased with gestational age from only 6.8 ± 1.4/FOV in very premature infants to 8.1 ± 1.7/FOV in moderately premature infants and 10.5 ± 0,8/FOV in term neonates (Figure 2A). Very recently, Nussbaum et al. similarly reported a gestational age-dependent increase of leukocyte adhesion in a dynamic flow chamber approach [6]. They also found comparable leukocyte adhesion when P-selectin is replaced by E-selectin [6].

To investigate the role of the integrins for leukocyte adhesion preterm infants and neonates, we compared leukocyte adhesion in flow chambers only coated with P-selectin and IL-8 with those with additional ICAM-1 coating. We found reduced leukocyte adhesion in ICAM-1 lacking flow chambers in all age groups, although the level of significance was not reached in premature infants (Figure 2B). This might be caused by the relatively small number of samples as well as the generally low level of adhesion in the premature groups. Nevertheless, our results point towards an important role of the interaction of β_2_-integrins with their ligands and suggest that the combination of all three adhesion molecules used is crucial for effective in vitro leukocyte adhesion not only in adults [11], but also in preterm and term infants.

To determine the role of RAGE for leukocyte adhesion in preterm infants and neonates we replaced the β_2_-integrin ligand ICAM-1 by sRAGE and in a first step performed dose-finding-experiments. In previous experiments, sRAGE coating with 4 μg/ml was successfully used to induce adult leukocyte adhesion [11]. sRAGE coating concentrations ranging from 0.5-20 μg/ml were tested in neonates in combination with P-selectin and IL-8 and revealed optimal leukocyte adhesion at 4 μg/ml sRAGE, too (Additional file 1: Figure S5). Since this coating concentration was exactly the same as for ICAM-1 it was used in all consecutive experiments. Notably, sRAGE concentrations higher than 10 μg/ml resulted in a decrease in leukocyte adhesion which might be due to inhibiting effects of free circulating sRAGE in the flow chamber as a result of sRAGE coating overdose [44].

Next, we found that sRAGE mediates leukocyte adhesion in both adults and during investigated stages of fetal life (Figure 2C). Similar to ICAM-1-coating, RAGE-coating in combination with P-selectin and IL-8 induced significant adhesion of neutrophils from infants and adults when compared to uncoated controls (not depicted). As observed for ICAM-1-dependent neutrophil adhesion, we also found that the number of adherent neutrophils significantly increased with gestational age, i.e. 7.8 ± 1.4/FOV in very premature infants, 9.4 ± 1.7/FOV in moderately premature infants and 11.6 ± 0.8/FOV in term neonates (Figure 2C, see also Additional file 1: Figure S4).

**Figure 3:**
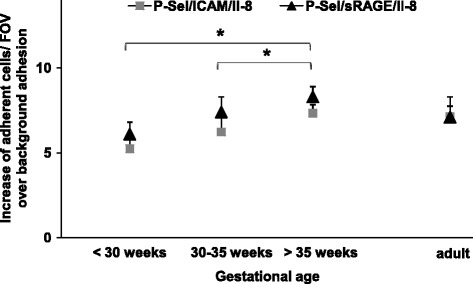
**Effective RAGE- and ICAM-1-dependent fetal neutrophil adhesion.** Neutrophil adhesion of preterm and term infants and adults was analyzed in dynamic microflow chamber experiments. The difference of neutrophil adhesion between uncoated controls and coating with P-selectin, IL-8 and RAGE or ICAM-1 is displayed as increase of adherent cells/FOV over background for the respective age groups. Results are presented as mean + SEM from at least 5 separate individuals/experiments per group. Significant differences (p <0.05) are indicated by asterisks.

As demonstrated and discussed earlier in this study, the preparation technique (separation or staining) cannot explain these observations. In order to rule out unspecific background adhesion inducing the increase of specific neutrophil adhesion during gestation, we first demonstrated that uncoated control neutrophil adhesion between investigated age groups is not statistically significant (Figure 2A). In addition, we calculated the effective neutrophil adhesion which is the increase of coated neutrophil adhesion over background (= coated-uncoated controls, Figure 3). We found that specific, P-selectin-, IL-8-, ICAM-1/RAGE-triggered neutrophil adhesion matures during gestation independently from background adhesion.

Thus, our results indicate that RAGE may be capable to mediate leukocyte adhesion similar to ICAM-1 not only in adults but also during early development. Former experiments in adult mice and humans have shown that RAGE may directly bind to the β_2_-integrin Mac-1 and thereby mediate leukocyte adhesion in-vitro and in-vivo [10,11]. In addition, we and others found that RAGE and ICAM-1 collaborate in mediating leukocyte recruitment in a stimulus dependent manner. As addressed later in these studies, RAGE predominantly binds to Mac-1, whereas ICAM-1 is the preferred ligand of the β_2_-integrin LFA-1 [10,11]. Up to date however, it is unclear whether these observations also hold true during fetal development. Moreover, only little is known about signaling properties of RAGE or its interaction with other ligands (i.e. HMBG1, S100) during early life [10,11,26-29].

Since RAGE, in contrast to ICAM-1, is highly expressed during early life [19], our key results of flow chamber experiments might also be of importance for the understanding of cellular immune defense mechanisms in inflammatory conditions during the neonatal period [2-8].

### Plasma sRAGE concentration during fetal development

**Figure 4:**
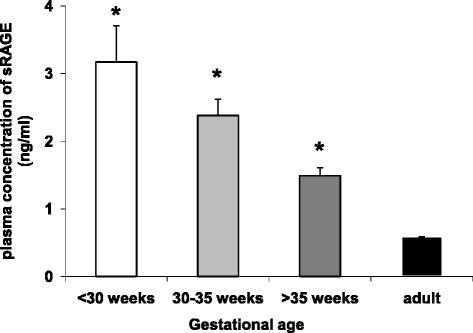
**Plasma sRAGE levels in adults and infants during late gestation.** The sRAGE plasma concentration of healthy adults was measured and compared to plasma of cord blood of neonates and premature infants (mean + SEM). Significant differences to adult levels are indicated by asterisk (p <0.05).

To test the hypothesis of high fetal RAGE expression in our study population, we measured the concentration of fetal sRAGE in the respective plasma samples of our preterm and term neonates and compared the results to adult sRAGE plasma levels. Indeed, we found that systemic sRAGE concentration is highest in infants born younger than 30 weeks GA and gradually decreases during the course of pregnancy (Figure 4). In term neonates, circulating sRAGE was only about 50% and during adulthood only 25% of the values measured in extremely immature infants. Cord blood sRAGE levels at this gestational age were higher than previously reported which might be explained by different techniques of detection [30]. Nevertheless, these findings point towards a distinct role of sRAGE for fetal life and a positive correlation between increased sRAGE and membrane RAGE expression during fetal development [20,21]. With regard to our flow chamber results we now argue that the role of RAGE-dependent leukocyte adhesion may be more prominent during early development than during later life. In combination with an increasing expression and role of ICAM-1 during fetal life [6] RAGE could also be crucial for the tightly regulated balance of fetal immunotolerance and cellular defense.

In a recent study of premature infants with funisitis, sRAGE was found to be decreased in cord blood and in the tracheal fluid [30], and there is increasing evidence that low sRAGE blood levels correlate with poor outcome in infants [21,45,46]. Although sRAGE has been additionally described to be associated with many other inflammatory conditions [28,30,31,47-49], it remains controversial whether a decrease in sRAGE levels is causative or a result of proinflammatory stimulation [25]. Therefore, the exact role of sRAGE for inflammatory conditions particularly during early development still needs to be determined.

### LFA-1 and Mac-1 expression during fetal development

We next investigated the age-dependent expression of the β_2_-integrins LFA-1 and Mac-1 which are known to be relevant receptors of ICAM-1 and RAGE.

**Figure 5:**
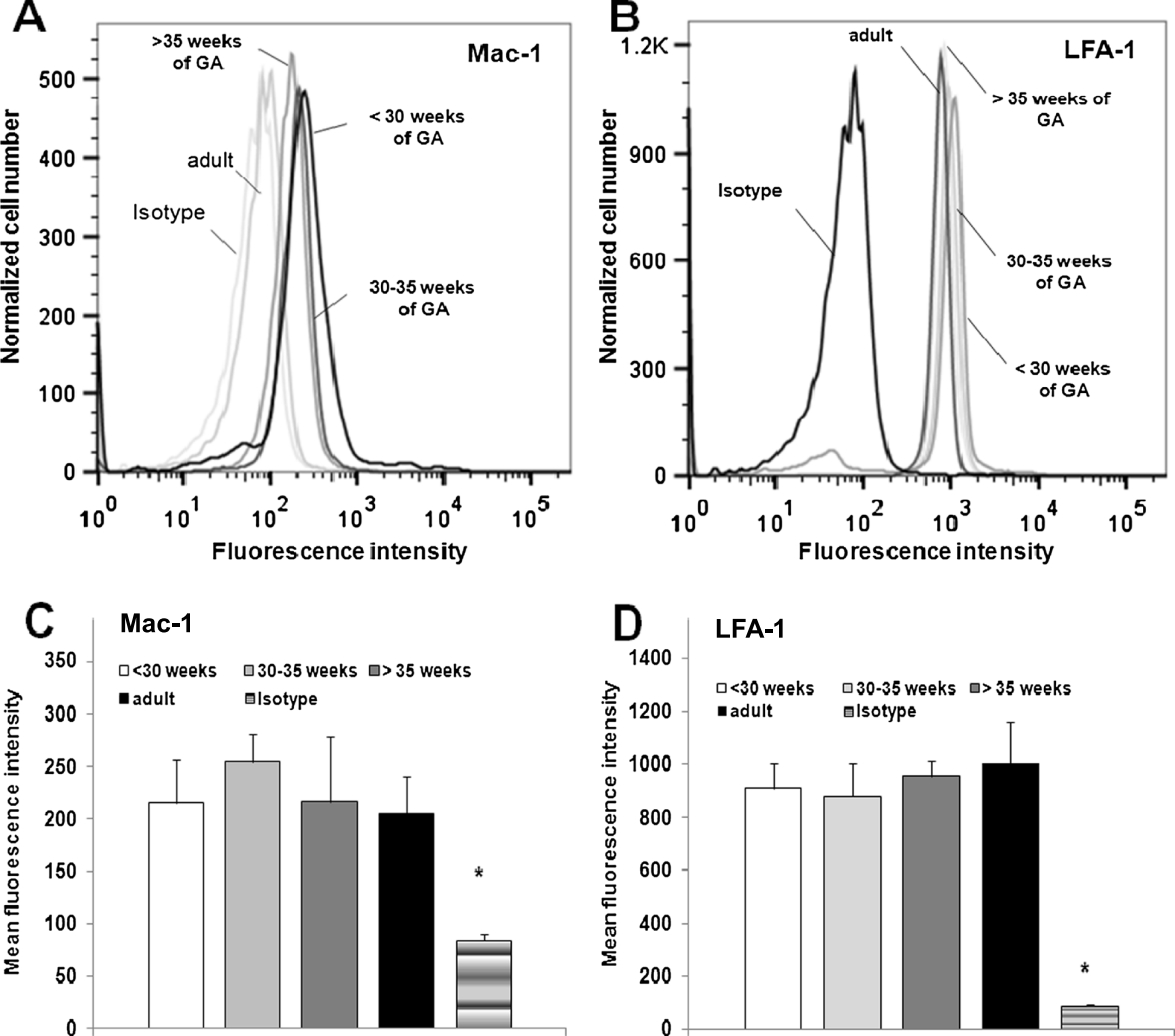
**Flow cytometric analysis of expression of LFA-1 and Mac-1 in adults and infants during late gestation. (A)** Representative Mac-1 and **(B)** LFA-1 expression on neutrophils isolated from cord blood of preterm and term infants of different gestational ages were compared to adults and respective isotype controls. Alternatively, bar graphs of the mean fluorescence intensity of Mac-1 **(C)** and LFA-1 **(D)** expression are depicted for the respective investigated groups. These results are presented as mean + SEM from at least 3 separate experiments/group. Asterisks(*) indicate significant difference vs all other groups at p <0.05.

Our flow cytometric investigations of isolated neutrophils revealed a constant LFA-1 and Mac-1 expression during early and late development (Figure 5A-D). In particular, neutrophils form premature and mature infants and adults did not show significant differences in LFA-1 and Mac-1 expression. The tendency of slightly lower Mac-1 expression in some adults (Figure 5A + C) might be attributed to faster isolation-induced Mac-1-upregulation in neonates (data not shown). These results are consistent with former studies which found similar levels of LFA-1 in preterm infants, neonates and adults, while expression of Mac-1 was reported to gradually increase during pregnancy in some studies [16,50], which might be explained by different experimental conditions.

We suggest, however, that the increase of ICAM-1-and RAGE-mediated leukocyte adhesion during early development is not primarily caused by altered fetal β_2_-integrin expression. Since expression may differ from function, maturation of LFA-1 and Mac-1 activity or avidity could be one explanation of our results. In addition, there are other leukocyte-born binding partners of the adhesion molecules coated in the flow chambers like PSGL-1 and IL-8 receptor [9]. Since PSGL-1 expression is reported to gradually increase with gestational age, while IL-8 receptor expression stays constant during fetal life [6], maturation of PSGL-1 expression can be another explanation of our findings. Moreover, fetal leukocytes could express other or unknown ligands of the coated adhesion molecules when compared to adult leukocytes. In this context one may also ask whether the recently observed homophilic RAGE-RAGE interaction [51] might take place between leukocyte- expressed RAGE and endothelial RAGE in the fetus. However, these questions have not been addressed so far and should be investigated in future studies.

## Conclusion

Our results suggest that impaired neutrophil adhesion of very premature infants only normalizes late during gestation. This study shows for the first time that RAGE controls leukocyte adhesion not only in adults but also in premature and mature infants. The pivotal importance of RAGE is supported by its high expression during fetal life. Thus, new therapeutic approaches for the treatment of inflammatory diseases of preterm infants and neonates could target RAGE, an approach which would most likely be relevant in non-infectious inflammatory diseases. The findings also expand our still incomplete knowledge of the fetal development of the innate immune system.

## Additional file

Additional file 1: Figure S1. Representative May-Gruenwald staining of cell suspension after PMN isolation of cord blood of a term infant. Bar represents 25 μm. **Figure S2.** Forward/sideward scatter dot plot of flow cytometric analysis of cell suspension with 10^5^ cells before (A) and after (B) isolation of PMNs of adult whole blood (P1= neutrophils, P2 = monocytes, P3 = lymphocytes). After isolation (B) 9300 cells (93%) were detected in the neutrophil gate, <100 cells in the monocyte gate (<1%) and 300 cells in the lymphocyte gate (3%) while about 400 cells were outside these gates (4%). **Figure S3.** Forward/sideward scatter dot plot of flow cytometric analysis of cell suspension before (A) and after (B) isolation of PMNs of term infants > 35 weeks of gestational age (P1 = neutrophils,P2 = monocytes, P3 = lymphocytes and erythroid progenitors). After isolation (B) 8800 cells (88%) were detected in the neutrophil gate, <100 cells in the monocyte gate (<1%), while 1200 cells (12%) were in the lymphocyte gate or outside these gates. **Figure S4.** Representative screenshot of a recorded flow chamber experiment with leukocytes isolated from a term neonate flowing through a chamber coated with P-selectin, IL-8 and sRAGE after 10 min. Big arrow indicates flow direction. Small arrows indicate adherent leukocytes. White bar represents 30 μm. **Figure S5.** Neutrophil adhesion on flow chambers coated with P-selectin (4μg/ml), IL-8 (10μg/ml) and different concentrations of sRAGE is shown as mean + SEM from at least 5 newborns and experiments per concentration. *indicates significant differences (p < 0.05) vs 0μg/ml sRAGE.

## Abbreviations

FOV: Field of view
HELLP: Hemolysis, elevated liver enzymes and low platelet count
ICAM-1: Intercellular adhesion molecule-1
LFA-1: Leukocyte functional antigen-1
Mac-1: Macrophage antigen complex 1
PMN: Polymorph-nuclear leukocytes
RAGE: Receptor of advanced glycation endproducts
sRAGE: Soluble RAGE
