# Supplementary material for: RAGE controls leukocyte adhesion in preterm and term infants
Source: BMC Immunol. 2014 Nov 27;15:53. doi: 10.1186/s12865-014-0053-0 (PMC4256735; doi:10.1186/s12865-014-0053-0)
Supplement: Additional file 1: Figure S1. — Representative May-Gruenwald staining of cell suspension after PMN isolation of cord blood of a term infant. Bar represents 25 μm. Figure S2. Forward/sideward scatter dot plot of flow cytometric analysis of cell suspension with 105 cells before (A) and after (B) isolation of PMNs of adult whole blood (P1= neutrophils, P2 = monocytes, P3 = lymphocytes). After isolation (B) 9300 cells (93%) were detected in the neutrophil gate, <100 cells in the monocyte gate (<1%) and 300 cells in the lymphocyte gate (3%) while about 400 cells were outside these gates (4%). Figure S3. Forward/sideward scatter dot plot of flow cytometric analysis of cell suspension before (A) and after (B) isolation of PMNs of term infants > 35 weeks of gestational age (P1 = neutrophils,P2 = monocytes, P3 = lymphocytes and erythroid progenitors). After isolation (B) 8800 cells (88%) were detected in the neutrophil gate, <100 cells in the monocyte gate (<1%), while 1200 cells (12%) were in the lymphocyte gate or outside these gates. Figure S4. Representative screenshot of a recorded flow chamber experiment with leukocytes isolated from a term neonate flowing through a chamber coated with P-selectin, IL-8 and sRAGE after 10 min. Big arrow indicates flow direction. Small arrows indicate adherent leukocytes. White bar represents 30 μm. Figure S5. Neutrophil adhesion on flow chambers coated with P-selectin (4μg/ml), IL-8 (10μg/ml) and different concentrations of sRAGE is shown as mean + SEM from at least 5 newborns and experiments per concentration. *indicates significant differences (p < 0.05) vs 0μg/ml sRAGE. [file 12865_2014_53_MOESM1_ESM.doc]

**SUPPLEMENT**

**
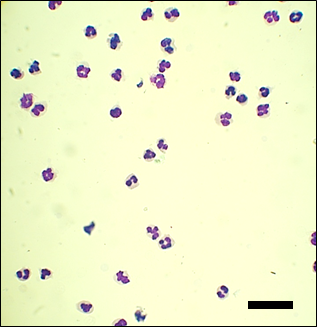
**

Supplemental Figure 1: Cell differentiation of PMN isolate in newborns. Representative May-Gruenwald staining of cell suspension after PMN isolation of cord blood of a term infant. Bar represents 25 µm.


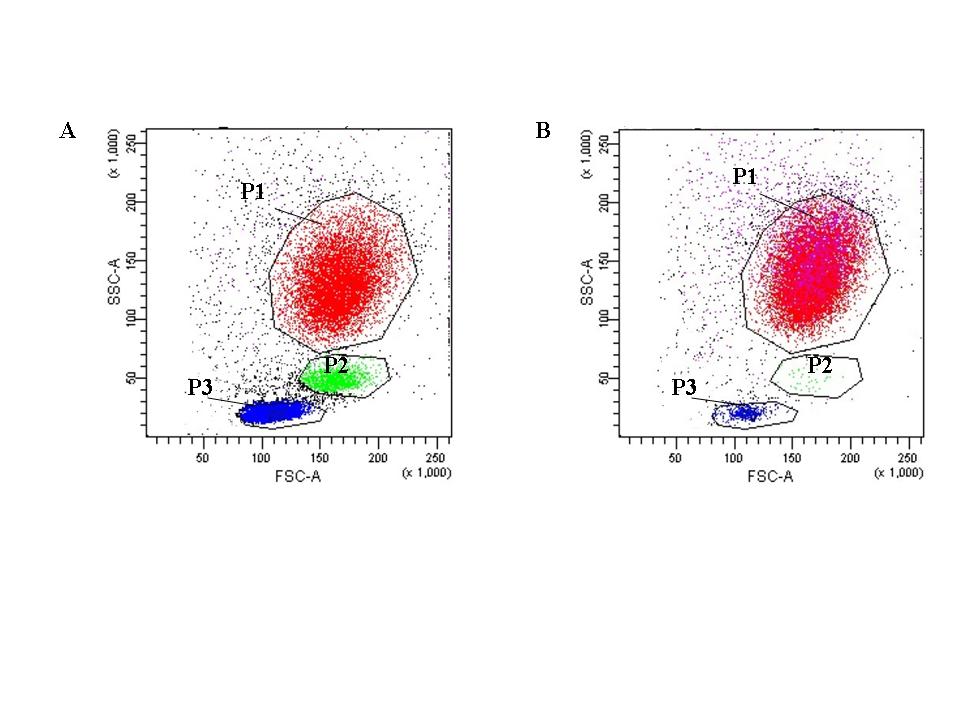


Supplemental Figure 2: Isolation dependent cell differentiation in adults. Forward/sideward scatter dot plot of flow cytometric analysis of cell suspension with 10^5^ cells before (A) and after (B) isolation of PMNs of adult whole blood (P1= neutrophils, P2 = monocytes, P3 = lymphocytes). Before isolation (A) 5550 cells were found in the neutrophil gate, 1100 cells in the monocyte gate and 2300 cells in the lymphocyte gate while 1150 cells were outside these gates. After isolation (B) 9300 cells (93%) were detected in the neutrophil gate, <100 cells in the monocyte gate (<1%) and 300 cells in the lymphocyte gate (3%) while about 400 cells were outside these gates (4%).


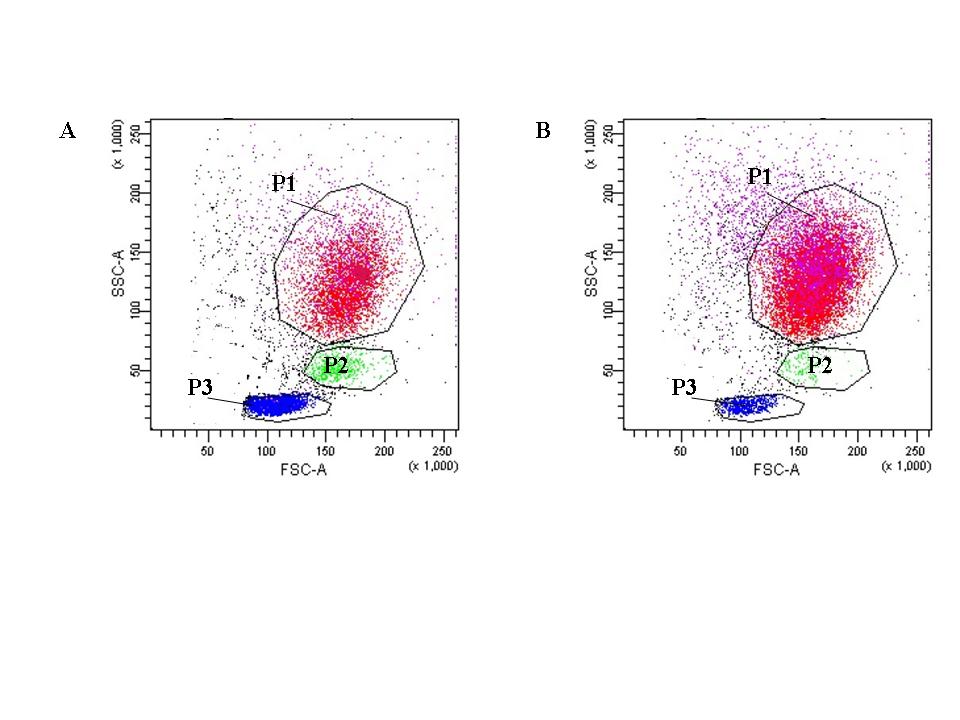


Supplemental Figure 3: Isolation dependent cell differentiation in newborns. Forward/sideward scatter dot plot of flow cytometric analysis of cell suspension before (A) and after (B) isolation of PMNs of term infants > 35 weeks of gestational age (P1 = neutrophils, P2 = monocytes, P3 = lymphocytes and erythroid progenitors). Before isolation (A) 4100 cells were found in the neutrophil gate, 600 cells in the monocyte gate and 3100 cells in the lymphocyte gate while 2200 cells were outside these gates. After isolation (B) 8800 cells (88%) were detected in the neutrophil gate, <100 cells in the monocyte gate (<1%), while 1200 cells (12%) were in the lymphocyte gate or outside these gates.


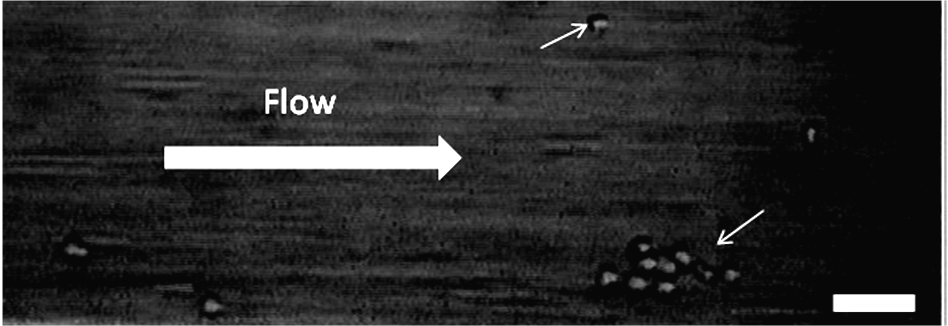


Supplemental Figure 4: Screenshot of a flow chamber experiment of newborns. Representative screenshot of a recorded flow chamber experiment with leukocytes isolated from a term neonate flowing through a chamber coated with P-selectin, IL-8 and sRAGE after 10 min. Big arrow indicates flow direction. Small arrows indicate adherent leukocytes. White bar represents 30 µm.


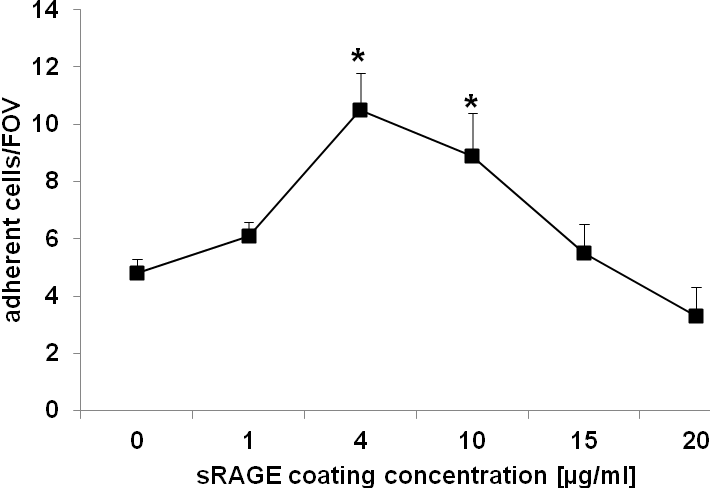


**Supplemental Figure 5: Neutrophil adhesion in sRAGE coating dose-finding flow chamber experiments of neonates.** Neutrophils isolated from term infants were perfused through microflow chambers coated with P-selectin (4µg/ml), IL-8 (10µg/ml) and different concentrations of sRAGE at constant flow conditions. Neutrophil adhesion per field of view is shown as mean + SEM from at least 5 newborns and experiments per concentration. * indicates significant differences (p < 0.05) vs 0µg/ml sRAGE.
